# eHealth Technologies, Multimorbidity, and the Office Visit: Qualitative Interview Study on the Perspectives of Physicians and Nurses

**DOI:** 10.2196/jmir.8983

**Published:** 2018-01-26

**Authors:** Graham G Macdonald, Anne F Townsend, Paul Adam, Linda C Li, Sheila Kerr, Michael McDonald, Catherine L Backman

**Affiliations:** ^1^ Arthritis Research Canada Richmond, BC Canada; ^2^ Rehabilitation Sciences University of British Columbia Vancouver, BC Canada; ^3^ Psychology Applied to Health University of Exeter Medical School University of Exeter Exeter United Kingdom; ^4^ Vancouver Coastal Health Vancouver, BC Canada; ^5^ Department of Physical Therapy University of British Columbia Vancouver, BC Canada; ^6^ Arthritis Patient Advisory Board Arthritis Research Canada Richmond, BC Canada; ^7^ W Maurice Young Centre for Applied Ethics School of Population and Public Health University of British Columbia Vancouver, BC Canada; ^8^ Department of Occupational Science and Occupational Therapy University of British Columbia Vancouver, BC Canada

**Keywords:** eHealth, patient-physician relationship, relational ethics, multimorbidity, online information seeking, email, office visit, health professional perspective

## Abstract

**Background:**

eHealth is a broad term referring to the application of information and communication technologies in the health sector, ranging from health records to telemedicine and multiple forms of health education and digital tools. By providing increased and anytime access to information, opportunities to exchange experiences with others, and self-management support, eHealth has been heralded as transformational. It has created a group of informed, engaged, and empowered *patients as partners*, equipped to take part in shared decision making and effectively self-manage chronic illness. Less attention has been given to health care professionals’ (HCPs) experiences of the role of eHealth in patient encounters.

**Objective:**

The objective of this study was to examine HCPs’ perspectives on how eHealth affects their relationships with patients living with multiple chronic conditions, as well as its ethical and practical ramifications.

**Methods:**

We interviewed HCPs about their experiences with eHealth and its impact on the office visit. Eligible participants needed to report a caseload of ≥25% of patients with multimorbidity to address issues of managing complex chronic conditions and coordination of care. We used a semistructured discussion guide for in-depth interviews, and follow-up interviews served to clarify and expand upon initial discussions. Constant comparisons and a narrative approach guided the analyses, and a relational ethics conceptual lens was applied to the data to identify emergent themes.

**Results:**

A total of 12 physicians and nurses (6 male, 6 female; median years of practice=13) participated. eHealth tools most frequently described were Web-based educational resources for patients and Web-based resources for HCPs such as curated scientific summaries on diagnostic criteria, clinical therapies, and dosage calculators. Analysis centered on a grand theme of *the two-way conversation* between HCPs and patients, which addresses a general recentering of the ethical relationship between HCPs and patients around engagement. Subthemes explain the evolution of the two-way conversation, and having, using, and supporting the two-way conversation with patients, primarily as this relates to achieving adherence and health outcomes.

**Conclusions:**

Emerging ethical concerns were related to the ambiguity of the ideal of empowered patients and the ways in which health professionals described enacting those ideals in practice, showing how the cultural shift toward truly mutually respectful and collaborative practice is in transition. HCPs aim to act in the best interests of their patients; the challenge is to benefit from emergent technologies that may enhance patient-HCP interactions and effective care, while abiding by regulations, dealing with the strictures of the technology itself, and managing changing demands on their time.

## Introduction

eHealth is a broad term referring to the application of information and communication technologies in the health sector, ranging from health records to telemedicine and multiple forms of health education, support, and tools [1]. It has been described as a general orientation toward exploring the possibilities of information and communication technology in health [2]. By providing increased and anytime access to information, opportunities to exchange experiences with others, and self-management support, eHealth has been heralded as transformational. It has the potential to create informed, engaged, and empowered “patients as partners,” equipped to take part in shared decision making and effectively self-manage chronic illness [3].

eHealth has been championed as a way to coordinate care among professionals and advance the move away from a disease-centered model of care toward patient-centered approaches better suited to address the needs of patients with multiple chronic conditions [4]. It is thought that eHealth can correct for some of the shortcomings of the health care system that have frustrated patients and caregivers. These include long wait times for appointments and limited access to health care professionals (HCPs), poor communication with and between HCPs, and the challenges that come with managing multiple health conditions (multimorbidity) [5]. However, the changes that various eHealth technologies bring to relationships—between patients and HCPs or between HCPs—have received comparatively little attention in the midst of the digital transformation [6].

Relationships between patients and HCPs are built through communication, and the way and means by which this communication occurs is in the midst of an upheaval precipitated by digital and information technologies. It has been postulated that eHealth technologies have the potential to improve communication between patients (those with multimorbidities in particular) and their health professionals [7]. For example, in a study of patient-physician communication through a Web-based eHealth portal, patients had the opportunity to create and share narratives outside of the allotted time of their consultations, and these narratives helped physicians develop a better understanding of their patients’ situations [7]. In a study on the use of a digitally mediated personal physician presence online, it was found that both patients and HCPs welcomed using the platform and benefited from Web-based interaction [8]. Lygidakis et al identified that HCPs’ perceived barriers to use were usually dispelled upon becoming familiar with the tools and suggest that training is key to addressing this issue [8].

It has been found that patients’ Web-based information seeking can have positive impacts on the relationship with their physicians [9], but only if the accuracy and quality of health information are good [10]. Similarly, Laugesen et al noted that high-quality information does not itself replace the need for a previous trusting relationship with the HCP [11]. Despite these benefits, a 2017 review of 41 papers and 2 chapters published in 2000-2016 regarding adoption of eHealth information and apps notes that “Mainstream medical practice has not yet adapted to the ubiquitous use of the internet by patients” [12]. There remains a need to better understand perspectives from HCPs regarding eHealth because virtually all patient-provider interaction and health information will eventually be mediated by eHealth technologies [13]. It is, therefore, important to examine the new ethical and practical concerns brought about by changes in the patient-provider relationship that technological advancement has already put in motion. In the previous phase of our project, we found that both patients and HCPs were aware of a changing dynamic in their consultations where patients were more informed but uncertain, leading them to have more questions for their HCPs [14]. In general, informed and engaged patients were less reliant on their HCPs and no longer saw them as the gatekeepers of knowledge, having begun to regard themselves as the experts on their condition. Although some HCP participants relayed benefits and anticipated a positive future as more eHealth tools were more widely used, others were reticent to adopt new technology. Findings suggested a need for a clearer understanding of why some HCPs were wary of changing expectations, responsibilities, and obligations arising from patients’ use of Internet, apps, and other eHealth tools. The objective of this paper was to examine how eHealth affects patient-HCP relationships, with particular attention on the office visit. We consider ethical and practical considerations from the perspectives of physicians and nurses.

## Methods

### Design

This analysis is part of a larger, two-phase qualitative study; the protocol is described in detail elsewhere [3]. Ethical review and approval were provided by the University of British Columbia Behavioral Ethics Review Board. The design for the overall study was informed by narrative [15] and constructivist grounded theory [16] approaches, using a relational ethics lens. Relational ethics addresses the ethical content and decisions implicit in everyday relationships and conversations [17]. This suited the overall study goal: to develop an understanding of how patients living with arthritis and multiple chronic conditions and health care professionals perceived the influence of eHealth technologies in managing chronic illness. Findings from the first phase using focus groups with patients and HCPs [14] informed a second phase of in-depth interviews. Here, we focus on the interviews with physicians and nurses about their experiences with eHealth and its impact on the office visit.

### Eligibility and Recruitment

Eligible participants were health professionals with at least 2 years of experience beyond their professional degree. They needed to report a caseload of ≥25% of patients with multimorbidity to address issues of managing complex chronic conditions and coordination of care. Participants were recruited by circulating notices to local clinics and medical rounds and by asking colleagues to share notices (word-of-mouth). Generalist and specialist physicians and nurses were purposively targeted, given their small numbers in the phase one (focus group) portion of the main study, so that we could explore the office visit in greater depth. Because the main study focused on patients with arthritis and at least one additional chronic condition, recruitment of specialist practitioners was directed to rheumatology and internal medicine.

### Interviews

We used a flexible, semistructured discussion guide for in-depth personal interviews, and follow-up telephone interviews served to clarify and expand upon initial discussions. The discussion guide was informed by findings from focus group discussions with patients and health professionals [3]. Interviews were primarily conducted by a sociologist member of the research team (AFT) experienced in qualitative health research; a research assistant trained in qualitative data gathering conducted interviews and follow-up calls with 3 participants to accommodate HCP schedules. The interview topic guide is appended (see Multimedia Appendix 1), but it should be noted that interviews were conversational in nature and items were not asked verbatim or in the order presented. Audiotapes were transcribed verbatim by a professional transcriptionist and identifying information (names, places) was removed to protect anonymity. Participants are referred to by pseudonyms.

### Data Analysis

A narrative approach [18] was taken to ensure that the rich and complex data of the individual interviews were understood and presented in a way that remained faithful to the narrative of the interview and not taken out of context. Using constant comparison method, transcripts of the interviews were read repeatedly by 2 authors (GGM and CLB) to achieve immersion and develop a sense of the whole [19]. This was followed by multiple close readings of the text to pick out keywords and ideas to be used as codes for further organizing the data, a process conducted by the lead author (GGM). This initial analysis documented first impressions and thoughts to begin developing a coding scheme to be applied more broadly to the data, supported by using QSR NVivo 10 software. Once the codes had been applied to transcripts, the coded material was sorted into categories based on how the different codes related to one another [20]. The process of coding and sorting codes into meaningful categories required multiple iterations to adequately explore the different possibilities within the data. As categories were developed by the lead author, they were discussed with the last author until agreement was reached on labeling key categories. To minimize bias and achieve clarity, preliminary codes and transcript excerpts were shared with team members to test impressions and assumptions and revise the coding framework. The categories were then analyzed through a relational ethics lens to identify emerging themes along the lines of what was being valued and what was at risk in the relationships described by participants [21]. Of the 7 research team members, 4 read all transcripts and 3 reviewed excerpts selected by the primary analysts (GGM and CLB) based on their relevance to the emerging themes. The team discussed iterations during the analytical process by email, in person, or by phone in scheduled team meetings, and by responding to draft reports of findings. Collectively, the team brought perspectives from patients, practitioners, and sociology, health services, and ethics researchers.

The final analysis is represented by a grand narrative (the two-way conversation, comprising 4 categories) and 2 small supplementary narratives related to email conversations and anticipating the future of eHealth. Finally, quotes were selected to illustrate and substantiate the final narratives shared here. Pseudonyms are used for anonymity.

## Results

A total of 12 HCPs participated, each giving an in-depth interview of approximately an hour (range 25-78 min, mean 51 min; 9 in person, 3 by telephone) and a 12 to 31-min (mean 23 min) follow-up interview by telephone. All participants practiced in urban settings, with some serving patients from rural and remote populations who traveled to the city to see them. The HCPs worked in hospital, clinic, and private office settings. Of the participants, 3 were general practitioners, 2 were registered nurses, 1 was a nurse practitioner, 3 were rheumatologists, 1 was a physician clinician-scientist, and 2 were rheumatology fellows. Out of the total 12 HCPs, 6 were male and 6 were female, the median age was 46 years, and their years of practice ranged from the final year of rheumatology fellowship to 29 years, with a median of 13 years (Table 1).

Participants mostly used eHealth information resources during their office visits, with a few other technologies less frequently (Table 2).

Web-based resources for patients were most common, with all participants using this in some form or another. A majority of participants (n=10) used Web-based information sharing or education sites for physicians (such as UpToDate.com), some used email to communicate with patients, and some used electronic record systems to which patients may or may not have access. A third of participants used digital diagnostic tools (apps or online) in their office visits with patients. An example of how eHealth tools and resources were accessed and used is Donna’s description of a professionally curated online site that she finds useful in her practice as a rheumatologist, as shown in Textbox 1.

**Table 1:** Participant characteristics.

Pseudonym	Profession	Gender	Years in practice
Arlene	Nurse	Female	27
Donna	Fellow	Female	2
Elise	Nurse	Female	25
Gabriel	Physician clinician-scientist	Male	29
Henry	Fellow	Male	<1
James	Family physician	Male	13
Jocelyn	Rheumatologist	Female	25
Luanne	Rheumatologist	Female	6
Martin	Family physician	Male	2
Miranda	Rheumatology nurse	Female	18
Peter	Family physician	Male	<5
Richard	Fellow	Male	Not provided

**Table 2:** eHealth tools and technology cited by participants.

Tool or technology	Used and discussed	Mentioned, not used
Monitoring apps, for example, to monitor blood pressure, weight, and physical activity	1	4
Web-based information/resources for patients	12	-
Web-based resources (curated sites) for health care professionals (HCPs), for example, diagnostic criteria, disease activity calculators, and medication dosage calculators	10	-
Web-based portals with patients to access personal medical records and test results	5	-
Videoconferencing for patient visits and telehealth consultations	1	4
Email with patients	5	-
Social media	4	7
Diagnostic tools/decision aids	4	1

**Example of how eHealth tools and resources were accessed (Donna’s description of a professionally curated online site).**

Donna: So a company has to pay to access and then essentially they’ve made a whole bunch of articles spanning all areas of medicine that are kept, as it says, “up to date.” So every 3 to 6 months someone goes in, looks at all the literature, and updates it. And they get people that are experts in that area to keep it updated. And so it’s an easy way to go to without having to go through all the literature yourself or go to your textbooks, which are now out of date to get a quick answer.

So if you are in the clinic and you’ve just forgotten the appropriate treatment for someone, you can look it up, which is handy when you’re still in the learning process. And it breaks things down into talks about the causes for the disease, different things to think about, all the medications, how you treat it, pros and cons, limitations of our knowledge, how to follow people.

It also has a drug calculator and it can tell you, you know, all the background information about medications, what the dose is if they have, say, an organ problem like kidney failure, if you have to dose adjust for that or whether or not you have to. It can tell you that. It tells you what to monitor, what to watch out for, and when not to use the medication. And you can also do a drug interaction. So if someone is on, for example, one of our medications, allopurinol, it often interacts with other medications. So you can put that in and then check to make sure there aren’t interactions before you prescribe it.

### The Two-Way Conversation

The underlying theme identified was labeled “the two-way conversation.” It concerns HCP-patient communication, describing the dynamics of a conversation that is in the process of change (regardless of how long the participant had practiced). Several participants described this as a shift toward a two-way conversation in medical visits, explaining how a more collaborative interaction with patients has evolved, in part facilitated by eHealth technologies.

We first explore the HCP perspective on the *evolution* of this two-way conversation through the rise of eHealth, and then examine their perspectives of the impact of eHealth on the present state of collaborative consultation: *having*, *using*, and *supporting* the conversation with patients. We present ways in which the two-way conversation is seen to facilitate the therapeutic relationship: how it is viewed as helpful to HCPs and how they try to support patients to be better able to engage in a two-way conversation.

### Evolution of the Two-Way Conversation

Participants described the historical relationship between patients and HCPs as having changed socially, technologically, and generationally from a relationship defined by a largely paternalistic power dynamic toward one based on collaborative partnership (Donna—Textbox 2).

They attributed this to the rise of the Internet bringing about widespread availability of information, meaning that patients were no longer relying solely on their HCPs for all of their information regarding their health (Luanne and Elise—Textbox 2). The Internet has facilitated this shift through providing the platform for patients to organize themselves in online communities where they can discuss their conditions, share favored resources, complain, or offer support to one another, connecting and empowering them (Jocelyn—Textbox 2). A characteristic of this more cooperative model is that more informed patients are more engaged and involved in making health decisions with their HCPs (Elise and Jocelyn—Textbox 2). These broad changes were regarded by participants as a generational shift in attitudes and ideas around health care practice among both patients and HCPs, to which HCPs of previous generations may have difficulty adapting (Peter—Textbox 2).

### Having a Two-Way Conversation

Overall, participants were enthusiastic about more informed patients as contributors to better health outcomes. There were a range of approaches to two-way dialogues described by participants, from “partnership,” emphasizing collaboration and teaching, to “alliance,” emphasizing patient choice. Martin embraced the idea of patients as “partners,” seeing a partner as someone “who simply helps me in bettering an outcome” by educating themselves and conscientiously monitoring their condition and behavior (Textbox 3).

Peter echoed this notion, stating frankly that patients who are engaged through eHealth and informed about their condition “are more useful, clinically” (Textbox 3). Peter related the usefulness of informed patients to their understanding clinical language that enhanced their ability to hold a “back-and-forth conversation” as opposed to “a one-way conversation where you would tell them” (Textbox 3).

The two-way conversation is more difficult when there are opposing views being expressed. Henry explains how he navigates these discussions, emphasizing the importance of maintaining the “therapeutic alliance” and treating patients as adults who “can make up their own minds” (Textbox 3). Gabriel (Textbox 3) outlined a similar approach, offering to discuss the scientific merits of different treatments with patients who came to him interested in trying alternative or non-Western medicine that they discovered on the Internet. Throughout their interviews, Henry and Gabriel used more paternalistic language to describe their interactions with patients than most participants, that is, suggesting or stating they knew what was in the patient’s best interest, but still stressed the importance of “letting” patients make their own decisions. They identified their medical expertise as the reason patients seek care, and subsequently used very direct terms to share their opinions. Henry and Gabriel took a hands-off, laissez-faire approach to patient education that placed greater importance on patient autonomy and less emphasis on the pedagogical role of the HCP.

**Evolution of the two-way conversation.**

Donna: Historically...physicians have been seen as someone not to question. [Patients from an older generation have] kind of come in and been more passive, in a sense, because theirs is more the paternalistic model, right, which is to do what the doctor says. Whereas the newer generation, it’s more as it should be: a collaborative effort.

Luanne: ...the whole paternalistic pattern is changing toward more patient centered and then more patient driven. But it has been facilitated by the Internet and the availability of information.

Elise: But patients were almost 100% reliant on what their physician or what their health care provider tells them. That was their primary source of information. And so now it’s very much more a team working together because they are coming with the information that they have sought from all sorts of resources and it’s usually the Internet. And, you know, sometimes they might be more up to date with the most recent research than you might be, for example, as a health care provider. So it really does become a team. And patients now, they don’t need to rely on their physician to give them the information they can find themselves. And I think it’s huge in that they can then make, and they are making, a lot more decisions for themselves about their health care.

Jocelyn: ...the Internet is what I think has enabled that change for a number of reasons. I think it allows people to not feel alone, to be able to more easily connect with other patients...It’s sort of like the strength in feeling like you’re part of a community as opposed to be isolated that I think empowers people to be able to do that. I think the Internet provides them with knowledge so that they’re not relying on their physician for the knowledge...There’s more than just knowledge. There are tools on the Internet which some people use and some people don’t that are empowering. So I think it empowers patients to make them arrive at the table more informed than in the relationship of dependence.

Peter: I think I’m part of a generation that we have kind of learned, we’ve been brought up with all this technology and so I think we, I’m not trying to put myself on a pedestal, but like I feel like we are much more equipped at using these tools as opposed to even my mentors who started practicing 30 years ago. I think it’s a lot easier for us to use this. Even if somebody, you know, a patient comes in throwing tools at you. I may better be able to handle that through the computer. We have more knowledge to discuss that with them as opposed to somebody who was, you know, trained 30 years ago. They might just deny it and get angry by that.

**Having a two-way conversation.**

Martin: Well, I want them to be my partner. I think outcomes are better and it’s certainly easier for me if the patient can do some of their own education and some of their own monitoring. Well just to help me out, I mean I have more patients than I can handle and so any bit of help is welcome.

Peter: ...it gives [patients] access to health information that they might not have had access to in the past...[they are] able to communicate their concerns and hopes and expectations and connect it with, you know, specific health. They are more useful clinically,...able to communicate what they want in a better, more effective manner with clinicians as opposed to it just being a one-way conversation where you would tell them. They’re able to do more of a back and forth conversation now. I guess you’d say that it’s empowering them more than it was before.

Henry: Well I think different people have different approaches. You know, at the end of the day, saying something and someone not hearing it and then saying it louder is certainly not like a solution. So if they’re sort of committed to a certain framework I think it’s important that I discuss the alternative. But, you know, you don’t want to damage the therapeutic alliance. So you propose your own perspective and provide your own resources. And beyond that, as long as they understood everything you said, they’re adults and they can make up their own minds.

Gabriel: And they went to this XYZ website. Usually it’s “What do you think about these natural remedies for this?” And I think that’s the ones that they come looking for alternatives that is not traditional Western medicine because they read all the side effects or they’re looking for validation of the natural alternative therapies...Well I say that pretty much in medicine that what we do these days is based on science and I’m not against that [alternative]. If they have good science we can discuss that and if it’s just based on no science then I can’t say. I just say, well you can try it if you want. [But]...the time that you’re going to lose before you’re getting proper treatment is valuable time.

James: We have to be people who not just have that information but we have the way to interpret it and we’re the ones to say, “Well you read about this study online or you read this thing on [X’s] website. Well, you know what, this actually hasn’t been shown or this was a rat study or there are actually studies that [show] this could be dangerous or this was a study done on post-menopausal women and you’re an 18 year old guy. And so this is how it may not be applicable.” So it’s really exciting because we can actually do more. The person is sort of coming in already thinking about things, forming questions and so I think it really helps us to perform health literacy in a much more meaningful and deeper way. And I think that there are patients that really value that.

James described a pedagogical approach to interacting with the eHealth users among patients (Textbox 3). In this view, the HCP understands “the way to interpret” Web-based information and has the responsibility to impart accuracy and applicability, and encourage patients to improve “health literacy in a deeper, more meaningful way.” He conveyed genuine interest and excitement with two-way conversations with informed patients. Other participants expressed similar notions, being enthusiastic about the ability of patients to do the research and assimilate complex information, but also holding that HCPs were the fail-safe for patients who often lacked the skills and tools to critically assess the information. Arlene talked about bringing a breadth and depth of knowledge to the conversation:

So what differentiates the really educated patient on a certain thing from someone who has trained in that? I suppose the main thing is that the health care professional is trained in multiple areas. So if someone becomes an expert in their own disease but they don’t know that disease and how it is [related] to other diseases.

### Using the Two-Way Conversation for Patient “Buy-In”

Participants spoke of valuing the two-way conversation as an opportunity to obtain “buy-in” from patients. When acknowledging shared decision making, the more prevalent description was of a conversation that focused on providing information or rationale for the recommended treatment. For example, Jocelyn mentioned the necessity of building patient understanding of their health conditions to improve treatment adherence (Textbox 4).

Richard and Donna saw this approach as essential for countering misconceptions about a medication that was frightening due to what the patient perceived as its “poor reputation” (eg, potentially harmful effects or undesirable characteristics) but from the HCP’s perspective had solid evidence as effective (Textbox 4). Jocelyn expanded on the topic of adherence to emphasize that empowerment of the patient through increased access to information and use of eHealth tools helps the HCP to build better relationships and understand patients—especially ones with multi-morbidities—more thoroughly and holistically (Textbox 4).

### Supporting the Patient to Engage in a Two-Way Conversation

The quality and quantity of information available to patients were the main concerns of all participants. They shared that the ability to have a productive two-way conversation relied on how patients used the Internet, apps, or decision tools.

Because it was easy to be overwhelmed by the amount of information some patients brought to the visit, most participants had developed management strategies. For example, Martin, a family physician, recommended specific websites he considered trusted sources, as did Luanne, a rheumatologist who emphasized the importance of valid websites to patients (Textbox 5).

The 3 nursing participants (Arlene, Elise, and Miranda) saw it as part of their job to spend more time directing patients to resources, to save the physician time. Miranda noted that when patients came in to the office visit informed but not necessarily with accurate information, she viewed her role to “steer them in the right direction” after inquiring about where they found their information (Textbox 5). Arlene initially “steers” patients away from blogs and chat rooms because of their emphasis on negative experiences that she thought might skew patient perceptions (Textbox 5).

**Using the two-way conversation for patient “buy-in.”**

Jocelyn: I think that if patients are more engaged in the decision and they understand why they are taking a medication and they bought into the decision, that they will have better adherence. So I think it’s kind of a by-product of that as opposed to a direct thing, it’s not so much that I think that the tool itself targets adherence. But I think that if they are more engaged and they feel more part of it, the main thing about adherence—and that’s what I discuss with them—is if they understand why they are taking a medication, they are way more likely to follow up with the instructions. It’s when they don’t really understand why they are taking it, then as soon as it’s inconvenient or they forget or they have a bit of a side effect then they’re less likely to be adherent.

Richard: [referring to decision tools] And I really liked the idea of education especially for methotrexate and different medications because adherence to therapy is a big issue, I think, with patients. And getting them to buy in on what we’re doing and why we’re doing it—that will definitely improve patient outcomes.

Donna: So yeah, definitely, the next time I come across a patient that really dislikes Methotrexate, I will be referring them [to electronic resource]. And it’s also probably a good thing to have anyways for them to know about and to be able to go use as a reference. Because some patients whom we prescribe medication, they don’t want to tell you. They’ve just met you and they don’t feel comfortable telling you that they don’t quite trust you yet or they don’t trust the plan. So they’ve left and you think that they’ve started on something and then they come back six months later and they haven’t started it. So I think having them go to a site can help educate but also increase the actual adherence to treatment.

Jocelyn: I think it’s more than just adherence to medications. I think if we have an engaged patient then we empower them to be able to really manage their disease a whole lot more efficiently. So I think we’ll have better outcomes because we’ll have better care that overall fits better with the patient. So that to me is, we’ll have a better relationship with the patient, we’ll have better management of things other than just the medications and the medical aspects of the disease, which is really important for the quality of life and the dealing with the person as a whole and the disability of the whole.

**Supporting the patient to engage in a two-way conversation.**

Martin: Patients are doing a lot of their own research now. Often they’ll come in and say, “What do you think of this that I read about? What do you think of that?” I must admit that some of the newer things they’re more aware of than I am. I tell them, you know, if you read something that just came out, you probably know more about it than I do. And they’re fine with that. They realize the speed of information. So I just really tell them that there are some reputable sites and there are some not reputable sites. So, you know, if you were on the Berkley site or the Mayo site or VGH site or Health and Welfare Canada, we trust those sites.

Luanne: So depending a little bit on what they’re in for, some of it is patient support. So Arthritis Society I will send them to, I have lupus patients for BC Lupus Society has got a support page. If they just want more general medical information I can also send them to places like the Mayo Clinic, Rheumatology Network as other reliable sources of information. And I try to stress with the patients that whenever they’re going to look for sources of information, they should make sure that it’s coming from a valid provider.

Miranda: I can’t say it’s often that they come in with accurate information. There is so much information and I think how people search is what pops up. And if you check with people briefly about where they found their information, I think that most people come in somewhat informed and then we can kind of steer it down the right path from that point onward or at least help them, guide them down the right path.

Arlene: I tend to try to steer people away from blogs at least initially or those chat rooms because often times the people that are on those are not people that have had positive experiences or are having a positive result. I think it skews their perspective of what is going on. But the power of the personal story is really far more riveting and convincing.

Gabriel: Yeah for me, for instance, the use of sites, I know patients when they come to you and you have to provide information they usually get shocked first to get a diagnosis and second to start treatment. And so I give them readings. I print some information for them and tell them if they have more questions to go to these sites and then you come back with me and we can discuss it if you want.

“Guiding” or “steering” patients toward certain resources was a strategy common to all participants, for purposes of ensuring patients had reliable information and to acknowledge time issues. For example, time can be saved in the visit if patients avoid unreliable websites; as Gabriel notes, recommending reliable Web-based resources for questions that arise outside the office visit helps patient learn about, and possibly emotionally adjust to, a diagnosis or new treatment (Textbox 5).

HCPs were concerned not simply about the quality of the information on the Internet but about the patient experiences while searching for answers. Richard remarked that patients are “often misled by the Internet” and that this “can cause a lot of anxiety.” Elise explained how the quantity of information on the Internet itself could be “incredibly overwhelming” to patients trying to make sense of their situation. The following story from Luanne also points to the fact that HCPs should check their own propensity for overwhelming patients with information and should instead opt for digestible and accessible formats:

It’s really simple and when I first saw it, I thought, oh dear patients are going to be offended because it’s a little, dumbed down a little bit. I have yet to have anyone complain about that. They’re all, like, oh a single piece of paper. I can read this. There are pictures. There are not too many words and it’s fantastic. And so I think, you know, knowing that there are Internet sites that will give you that extra information if you need it, I don’t feel bad giving them a single page suggesting places they can go and read more if they want to.

Although the two-way conversation comprised the central narrative, the analysis generated two smaller narratives related to HCP-patient interactions, as follows.

### Conversation Beyond the Consultation: A Role for Email?

Different views were presented regarding potential benefits and drawbacks to using email with patients (Textbox 6).

One of the main concerns was related to time constraints and how email could create imbalance in their workload. Martin remarked that “when it’s an office visit, I feel like there’s a finite amount of time to have a consultation.” There are clear time boundaries in the office visit, he notes, but with email, “it goes on and on.”

Richard believed there should be a “professional barrier” between HCPs and patients in regard to email because an expectation exists that messages can be answered immediately, but the reality remains that this is not possible either due to lack of time or the need for a face-to-face discussion. Richard also suggested that “if a doctor would answer every email from every patient, that’s more than a full-time job.” Jocelyn was wary of setting up expectations with email because of the round-the-clock responsibility that it could entail:

They’re expecting me to check and respond because maybe in the past I have responded quickly. I’ve set up expectations. So instead of going to the Emerg or going to a walk-in clinic, they wait for my answer. And then maybe I don’t and it was an urgent situation and you don’t seek care. So that scares me. So it’s the expected immediacy of response I think...I think once you’ve started responding quickly, it sets up an expectation and I wonder to what extent I’m then responsible.

Likewise, Elise (Textbox 6) expressed how the move to electronic records in her office necessitated that “we’ve had to become even more aware of the privacy regulations” and when it came to email with patients “we actually shouldn’t be doing it” because of privacy issues. James, however, seemed to have worked out a system of regulating his email exchange with patients to enhance rather than impede efficiency. James offered that email (sometimes combined with telehealth approaches) could be the best use of his and his patients’ time, given that some experienced mobility issues, lived in remote locations, or both, and not all consultations required a face-to-face visit to be effective.

**The conversation beyond consultation: a role for email?**

Martin: Yeah there’s no end to it really. That’s the problem is, you know, the patient will ask a question. I’ll reply and then they’ll reply. I just don’t, you know, when it’s an office visit, I feel like there’s a finite amount of time to have a consultation. And then, you know, both the patient and I say, “okay, time’s up for this appointment. If you want to talk some more, come again.” But that doesn’t happen with email. With email it’s just, it goes on and on.

Richard: I try not to do that [email] because I think there has to be some professional barrier between patients and their physicians; otherwise sometimes you need a little bit of time for things to evolve. And I can understand if patients are anxious and they want to know the answer now. But sometimes you need time. You can’t answer it now. Blood work takes a week to come back or, you know, this test takes a little bit of time. Sometimes symptoms take awhile before you can really tell is this, what direction are we taking and, you know, if a doctor would answer every email from every patient, that’s more than a full-time job.

Elise: And, you know, since we—in one of the offices I work in—since we’ve gone from paper to electronic charts, we’ve had to become even more aware of the privacy regulations and, you know, I’m not sure how but it was brought to the office’s attention that emailing is, that we actually shouldn’t be doing it…you know, patients love it as it’s so it’s easy for them, right? They don’t have to worry about waiting for someone to answer the phone. You know, they can send it at midnight if they are thinking about something. But it’s really, we shouldn’t be doing it.

James: I mean many, the majority of these consultations are for people with whom I’ve had face-to-face consultations and we’ve made a plan. But to carry out that plan I don’t necessarily need them in the same room. And maybe part of the plan is that they have difficulty getting in to see me because of some of the barriers that we talked about. And for someone to come in and just show me the blood pressure readings that they’ve gotten, that’s not a great use of their time. And if they’re able to email it to me and then we’re able to talk about it, it’s efficient use of their time. It’s efficient use of my time and the patients really enjoy it.

**Looking toward the future of eHealth.**

Richard: I think that’s an area of opportunity...I think it would be great if patients had copies of their health records on some app, had copies of their imaging. I think that would be very useful. If we had a centralized system that would be useful. Like having [Hospital A, Hospital B, and Hospital C] in different systems is ludicrous, I think.

James: So I mean like anything health IT is a tool. And it has to be used properly. And if we go back to the analogy of the stethoscope, just because someone has a stethoscope doesn’t mean that they’re going to hear the murmur or they may mistake something to be pneumonia when it’s congestive heart failure. So the mere fact that having a stethoscope doesn’t mean that someone is going to use it properly and make good decisions about it unfortunately. And so the same thing holds here in that someone may use the tool, for example, video teleconferencing and see a patient online in a way that’s not productive or maybe for a complaint that isn’t appropriate or something that does need to be seen in person. So I think it’s important that as we gather experience and see what the limits are, those things will become clear and people will get together and there will be more guidelines. But right now we’re really on the cutting edge of people saying, hmm, okay we have this technology. There’s a potential for abuse but a potential for great benefit. But what we need to do is say, okay, are there guidelines that apply to everyone using it? Are there guidelines that apply to some people using it and some sorts of patients? And what parameters can we put on so that we’re using this to the best of our abilities?

### Looking Toward the Future of eHealth

Some participants spoke of the potential of eHealth tools and technology not yet routinely used in their practice. For example, they envisioned improved or new tools as paving the way toward more efficient team care by making coordination between HCPs easier, as in this example from Elise:

So many patients come in and they say, “Well I’m on some kind of blood pressure medication and I don’t know what it’s called.” So we can access that now from the office and you can find all of their medications [with patient consent, on the provincial network that records all prescriptions dispensed]. So in that way it’s a huge safety, you know, protection for the patient because you then know interactions and things like that…Then if a patient is seeing multiple specialists, which they often are..., they should all have access to everything, right? So it’s coming. It’s not there yet. We still spend quite a bit of time phoning over to a specialist’s office and saying, “Can we get that consult?” Because unless they specifically share it when they’re doing their dictation, we’re not going to see it, right?

The HCPs in our study described feeling burdened with the inefficiencies of the still-developing eHealth systems they are working within, in particular the electronic health record. It was declared that adequate resources are not in place, systems are not interoperable, and patient concerns and needs may outstrip what HCPs can provide (Richard— Textbox 7).

As a result, they expressed a need for improvements and innovation to ensure the systems they work within were truly functional. James (Textbox 7) made the case that although there may be many tools available, there is no guarantee that those tools will be used effectively and that guidelines drawn from experience should increase awareness of the capacity of eHealth to support practice.

## Discussion

### Principal Findings

The HCPs in our study saw eHealth technology as an important contributor to how their relationships with patients have changed and are evolving toward more collaborative care—both collaboration with the patient and with other providers. Participants expressed a desire for guidelines for how patients and HCPs communicate and use eHealth technology to optimize benefits and prevent undue burden or abuse. There were participants who were enthusiastic about emailing patients, and others had serious reservations about this. However, there was agreement that if this mode of communication was to work, it would need to be on the terms the HCP could manage in the context of their practice, which also included adhering to privacy regulations (the mechanisms of which were not always well understood by HCPs). Participants were concerned about how Web-based information, health apps, and email with patients could create unrealistic expectations for patients and unsustainable responsibilities for themselves in terms of workload and time.

Participants said they valued informed and engaged patients and saw patient engagement in health care as facilitated by eHealth, leading to better health outcomes. In general, the emergence of eHealth resources was regarded as a positive progression, despite hiccups and shortcomings. Though HCPs no longer see themselves as the gatekeepers of medical information, they described themselves as best able to translate that information for the benefit of their patients. Indeed, the most universal use and discussion of eHealth among HCPs in this study focused on how patients access health-related information on the Internet, with attention given largely to guiding patients toward higher-quality information and better understanding of that information. In describing their consultations with patients, participants seemed to adopt the discourse of collaboration, engagement, and empowerment. They were clearly caring and compassionate, yet elements of a paternalistic relationship with patients were evident in some descriptions. As outlined below, these findings raise important ethical issues.

The ways in which HCPs described how eHealth facilitated engagement and empowerment of their patients offer a window into a more nuanced understanding of shifting relational dynamics and practice ideology in a period of transition. Importantly, our participants did not view patient engagement or empowerment resulting from the rise of eHealth as eroding or taking away their power as HCPs. The engaged or empowered patient was described as an actor in the relationship who could more meaningfully participate in their care. The “two-way conversation” integrated eHealth tools and Internet information used by patients and HCPs, and was variously described as a way to teach patients about their illnesses, facilitate understanding of and adherence to medical advice, support how patients self-manage their chronic diseases, and enhance clinical effectiveness or efficiency.

Some descriptions of teaching patients evidence residual paternalism in that the two-way conversation, as described by some HCPs, did not denote a symmetrical flow of information, but rather cast the engaged, empowered, knowledgeable, and tech-savvy patient as more receptive to medical advice and open to the medical worldview. Most telling, perhaps, is the insight that HCPs were interested in how supporting patients to be engaged and empowered could facilitate “buy-in” or concordance with the HCPs’ perspective and judgment.

From the perspective of HCPs, patients were historically treated as subjects upon whom health care was practiced; the eHealth revolution has accelerated the patient-centered care philosophy and facilitated a relationship wherein the patient is a person who meaningfully participates in the process of his or her own treatment. The emergence of this new relationship between patients and HCPs is, however, far from complete, and it is unclear whether the role of eHealth will be to facilitate further changes in power dynamics or maintain the hierarchy between patients and HCPs [22]. Supporting patient engagement is not an either-or commitment but has different forms along a continuum: from consulting with patients and sharing information with them about a diagnosis, to involvement and asking them about their preferences for their plan, to partnership and sharing leadership and decision making [23]. Our findings suggest that there remains a tendency among some HCPs to view patient engagement and empowerment narrowly as a clinical tool rather than a collaborative endeavor.

Although participants candidly responded to questions, in interpreting these findings, it is reasonable to reflect on the research interviews as a social encounter where participants may feel pressed to respond in certain ways to present as current and knowledgeable professionals. Ambiguity can be detected between some of the ideals expressed and the way practices were described. For example, all participants genuinely conveyed a desire to achieve the best possible health outcomes for their patients and welcomed the notions of patient-centered care, collaboration, and shared decisions, primarily in the form of generic descriptions. Yet, descriptions of actual patient encounters that cite adherence and “steering” patients in the right direction show how challenging it can be to fully develop mutually respectful relationships that connect with the experience of what it is like to be a patient. HCPs’ work was also restrained by the practice environment, creating tension between ways they prefer to practice and ways they had to practice due to suboptimal systems. These observations have practical implications for reflective practice, such as encouraging HCPs to consider their goals for patient-centered care and how their practices, attitudes, and word choice may be (mis)interpreted by patients.

### Comparison With Prior Work

A study by Laugesen et al indicating that the quality of Internet health information has some impact on patients’ concordance with their physicians is further borne out by the practical concerns of our participants in guiding their patients to certain Internet resources over others [11]. Our participants also drew the connection between the quality of health information accessed by patients and patient compliance, which agrees with the finding of Laugesen et al that there is an indirect relationship between the two [11]. The changes in patient-HCP communication brought about by eHealth were heralded by participants as opportunities to teach and communicate with patients that could increase quality of care and lead to better outcomes, and taken together with the findings of Haskard-Zolnierek and Di Matteo imply that if eHealth technologies do indeed facilitate better HCP communication, they will likely facilitate better adherence as well [24].

From the standpoint of relational ethics, which locates the practice of ethics in the dynamics of everyday relationships, respect for the lived experience of patients is a basic requirement of ethical engagement [17]. Many of our participants either supported or advocated patient empowerment, but referred to this concept in concert with constructs that implied a hierarchy in decisions about care, such as “adherence.” Adherence was valued because it was associated with better outcomes, from a medical standpoint. Following Ajoulat et al, ethical issues may arise where HCPs too narrowly interpret what empowerment is and means to their patients, leading to other aspects of the illness experience being overlooked [25]. An attendant ethical issue often lies with a blurring of the distinctions between having responsibility and having power. Leveraging patients’ high level of engagement with eHealth to transfer onto them the responsibility for aspects of their health care will by no means necessarily result in their empowerment, as it is entirely possible to have responsibility without having power.

Our findings are in agreement with Kreps and Neuhauser [26], who have noted that eHealth technology, through providing timely, accurate, and accessible information to all stakeholders, can support decision making that meaningfully involves patients and providers, but only if they are interoperable and tailored to engage the personal context of patients. Furthermore, our participants agree that the holistic needs of patients with chronic illness demand technologies that facilitate a person-based rather than disease-based model of care [4]. Tools such as online patient diaries that bring the patient experience closer to the HCP [7,8] have the potential to help create the conditions for office visits to incorporate the relational ethics of person-centered care into practice by creating the space for a more effective meaningful exchange of information and negotiation and support of behavior change between patients and HCPs. Some of the frustrations with systems, such as electronic medical records that were not integrated across institutional boundaries, or worries about being inundated with requests if electronic communications were opened up to patients suggest an element of moral distress. Austin [27] described how environmental constraints contribute to moral distress, such as lack of time or structures to communicate with other team members or engage in problem solving or building relationships. Future research could explicitly examine frustrations as an indicator of moral distress to develop potential solutions to support practice.

Collectively, our participants used a relatively narrow range of eHealth tools and solutions, focusing primarily on health information, literacy, and email. Participants with a broader range of experience may raise different issues. However, based on prior studies [12], they may be typical practitioners and findings may inform health professional education specific to eHealth [12], better equipping HCPs to adopt eHealth technologies to strengthen patient-HCP relationships. Twelve respondents is a relatively small number, but issues were repetitive across participants, even though they had differing years of practice, experiences, and familiarity with eHealth, and differing descriptions of communication and caregiving styles. Thus, responses may be transferable to other physicians and nurses in office and clinic practice environments. Nevertheless, as eHealth technologies continue to evolve, examining their impact on effective patient-HCP relationships and health care decisions warrants investigation using different research designs, with larger, more heterogeneous samples that include populations with low health literacy and limited access to eHealth technologies.

### Conclusions

HCPs are at the forefront of dealing with the everyday ethical issues emerging from the growing role that eHealth technologies are playing in health care consultations. Their task is simultaneously to use emergent technologies to enhance their interactions with patients and facilitate a beneficial involvement in their health care, all the while abiding by the regulations of existing health care institutions, dealing with the strictures of the technology itself, and trying to manage the changing demands on their time. Potentially, eHealth supports the evolving nature of the reciprocity of the patient-HCP relationship, toward patient-centered care, enhanced communication, and efficient health service delivery.

## Appendix

Multimedia Appendix 1 Interview guide.

## Abbreviations

HCPs: health care professionals
