# Supplementary material for: eHealth Technologies, Multimorbidity, and the Office Visit: Qualitative Interview Study on the Perspectives of Physicians and Nurses
Source: J Med Internet Res. 2018 Jan 26;20(1):e31. doi: 10.2196/jmir.8983 (PMC5807622; doi:10.2196/jmir.8983)
Supplement: Multimedia Appendix 1 [file jmir_v20i1e31_app1.pdf]

Exploring E-Health Ethics & Multi-Morbidity

Interview Guide 1: HCP Face-to-face Interview

Introduction

The purpose of the interview is to get your perspective on and experiences of e-health; e.g. any Internet based technology, decision-making tools, monitoring devices, social media that you/your patients use or that you know of.

1. What types of e-health do you use in clinical practice?

Probe for:

Devices, types of e-health e.g. social media, decision tools/patient support applications/Skype/e-mail

How often/routinely do you use it/them (refer to their response)?
Probe about occasions when useful.

2. What role overall would you say that e-health plays in your clinical practice?

Probe for how it has changed things regarding:
- Challenges to practice
- Supporting practice

3. I want to talk in more detail now about Patient-HCP relationships

What role if any does e-health play in consultations with your patents?
Can you give me examples?
Probe for different types of patients e.g. combination of conditions/new or established diagnoses
How far does it support shared decision-making?
- Probe for their definition of shared decision-making.
- Does it introduce any challenges to shared decision-making?

How far does it support patients to prepare for the consultation?
How far does it change traditional roles/responsibilities/notions of trust?
Probe for examples
How far does it support teamwork?
Given your use of e-health what are the/would be the qualities in your doctor/HCP that you would/most value?
Has your view of patient/doctor/HCP relationship or roles changed over time (ref to e-health use) - if so how?

4. More general questions:

Thinking about the implications of e-health for the future:

- Can you seen any challenges/tensions
- What would you see as the benefits/potential for e-health?
- What kind of changes do you envision as technology advances?
- What do your experiences suggest?

5. Ethical issues (e.g. autonomy, harms)

Now shifting to ethical issues in particular:

- In your experience how far does e-health support patients to manage their conditions the way they would like?
- How far do you feel it supports them to make decisions about you regarding:
  - -Living with their conditions/activities?
  - -Medications?
  - -Other illness related decisions?
- Probe for role of e-health technology in the shifting balance of relationship.
  - o Might it introduce tensions/discord into consultations?
  - o Thinking about the role of e-health, what would be the ideal consultation/relationship with your patient?
- More generally – how important is it for everybody to have access to e-health? In what ways? (this gets at strengths/benefits, therefore probe for…) is there a 'down side' to e-health as you know it?

6. Before we finish is there anything else you would like to talk add/ask me?

7. What made you take part in this study?
